# Two “Edges” in Our Knowledge on the Functions of Ribosomal Proteins: The Revealed Contributions of Their Regions to Translation Mechanisms and the Issues of Their Extracellular Transport by Exosomes

**DOI:** 10.3390/ijms241411458

**Published:** 2023-07-14

**Authors:** Anastasia Ochkasova, Grigory Arbuzov, Alexey Malygin, Dmitri Graifer

**Affiliations:** Institute of Chemical Biology and Fundamental Medicine, Siberian Branch of the Russian Academy of Sciences, 630090 Novosibirsk, Russia; asgrosheva@gmail.com (A.O.); grigory_was@inbox.ru (G.A.); malygin@niboch.nsc.ru (A.M.)

**Keywords:** ribosomal proteins, eukaryotes, translation, extracellular transport of ribosomal proteins

## Abstract

Ribosomal proteins (RPs), the constituents of the ribosome, belong to the most abundant proteins in the cell. A highly coordinated network of interactions implicating RPs and ribosomal RNAs (rRNAs) forms the functionally competent structure of the ribosome, enabling it to perform translation, the synthesis of polypeptide chain on the messenger RNA (mRNA) template. Several RPs contact ribosomal ligands, namely, those with transfer RNAs (tRNAs), mRNA or translation factors in the course of translation, and the contribution of a number of these particular contacts to the translation process has recently been established. Many ribosomal proteins also have various extra-ribosomal functions unrelated to translation. The least-understood and -discussed functions of RPs are those related to their participation in the intercellular communication via extracellular vesicles including exosomes, etc., which often carry RPs as passengers. Recently reported data show that such a kind of communication can reprogram a receptor cell and change its phenotype, which is associated with cancer progression and metastasis. Here, we review the state-of-art ideas on the implications of specific amino acid residues of RPs in the particular stages of the translation process in higher eukaryotes and currently available data on the transport of RPs by extracellular vesicles and its biological effects.

## 1. Introduction

Ribosomes are a form of highly organized molecular machinery that perform the final stage of the realization of genetic information by translating it from the nucleotide sequence of messenger RNAs (mRNAs), copied from protein-coding genes in DNA, to the sequences of amino acid residues of proteins. Translation occurs according to the almost universal genetic code, which establishes correspondence between trinucleotide codons of mRNA, carrying the genetic information, and the amino acid residues. Ribosomes in eukaryotes comprise four molecules of ribosomal RNA (rRNA) and 79–80 ribosomal proteins (RPs) that are combined into two subunits, the small (40S) and the large (60S), one assembled around the 18S and 28S (25S in yeasts) rRNA, respectively. The subunits are united into the 80S ribosome by a dozen so-called intersubunit bridges formed by the components of both subunits. The small subunit forms an mRNA binding channel that accommodates about 30 nucleotides of mRNA during its translation. The large subunit contains the peptidyl transferase center (PTC), which catalyzes peptide bond formation between amino acid residues delivered to the ribosome by transfer RNAs (tRNAs) as aminoacyl-tRNAs. Additionally, the ribosome has three tRNA binding sites: A (aminoacyl, for incoming aminoacyl-tRNA), P (peptidyl, for tRNA with nascent peptide chain) and E (exit, for the binding of discharged tRNA before it leaves the ribosome) sites. In ribosomes from all domains of life, these sites are formed by both subunits so that anticodon stem-loops of tRNAs reside in the small subunit, where mRNA codons interact with the anticodons of cognate tRNAs. The acceptor stems of tRNAs charged with amino acid residues are placed in the PTC region of the large subunit (e.g., see [1,2,3]). Ribosomal proteins (RPs) are essentially implicated in the work of the ribosomal machinery and are directly and/or indirectly involved in the organization of all its functional sites. In the case of the PTC, their participation is indirect, since it is formed solely from nucleotides of the 25S/28S rRNA that are highly conserved in all domains of life (e.g., see [4]). However, the PTC structure is functionally competent due to a network of interactions between the rRNA and RPs, without which 23S/28S rRNA has no catalytic activity (e.g., see [5]). As for the mRNA binding channel and tRNA binding sites, RPs are directly implicated (together with rRNA) in their organization and make specific contacts with the respective translation participants, and thereby they are expected to directly participate in the translation process.

RPs that are located at a several-angstrom-long distance from the mRNA or tRNAs in the ribosome are regarded as contacting them; these RPs have been at first determined by site-directed cross-linking in model complexes of mammalian ribosomes with the application of various types of mRNA and tRNA analogues bearing cross-linker groups at certain locations [6,7,8,9,10]. Further studies enabled the identification of the RPs’ amino acid residues cross-linked to mRNA nucleotides in definite positions relative to the first nucleotide of the P site-bound codon [11,12,13] and to the 3′ terminus of deacylated tRNA at the P site in the hybrid P/E state [14]. After structures of the eukaryotic ribosomal complexes were deciphered at a near-atomic level via X-ray crystallography [15,16,17,18] and high resolution cryo-electron microscopy (cryo-EM) (e.g., see [19,20,21]), the data obtained via site-directed cross-linking were completely supported (e.g., for review, see [22]).

However, in spite of detailed knowledge on the structural organization of the ribosomal functional sites and contacts between RPs and mRNA and tRNAs, it is difficult to predict the particular contribution of each amino acid residue implicated in the formation of these sites or contacting ribosomal ligands to the translation process in living cells. In other words, it is not clear how translation will cause (if at all) changes in amino acid residues suggested to contact ribosomal ligands. These issues have been addressed in more recent investigations of cells, in which target RPs contained substitutions or deletions of amino acid residues that had been found earlier in close proximity to ribosomal ligands [23,24,25,26,27,28], near the PTC [29] or within an intersubunit bridge [30]. After estimating the step of translation or its regulation affected by the alterations inserted in the amino acid sequences of RPs, it turned out that the effect of the alteration could not be the one easily predictable from the structural studies. As well, the importance of peculiar amino acid residues of RPs, located in the functional sites of the ribosome, for the translation process was rather variable. Some of them were found to be critically essential for the translation process; the contribution of other amino acid residues of RPs to translation was moderate or associated with fine regulation of the process.

Currently known extra-ribosomal functions of RPs have been rather well-studied and summarized in a number of reviews (e.g., see [31,32,33,34,35]). For all that, mechanisms of the realization of such functions are known in less detail than those related to translation. The least understood and most poorly reviewed aspect of the life of RPs outside the ribosome is their participation in communication between cells as passengers of extracellular vesicles (EVs), including microvesicles, exosomes, etc. In recent years, the growing line of evidence for the presence of a number of RPs within such vesicles has accumulated (e.g., see [36,37,38,39,40]). Particular interest has caused data on the enrichment of certain RPs in EVs to be derived from malignant cells [41,42,43,44,45,46]. Moreover, it has been demonstrated that extracellular delivery of specific RPs by EVs can change the phenotype of the receptor cells so that it gains sensitivity to anti-cancer drugs [47] and/or enhance their malignant features [43,45]. 

**Figure 1:**
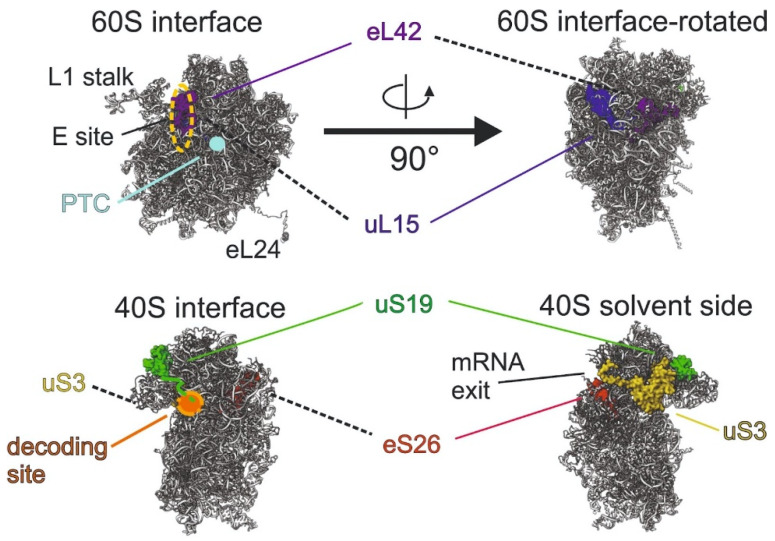
Ribosomal subunits of the mammalian ribosome (PDB: 4UG0), on which ribosomal proteins of interest are shown together with selected features of the subunits and the locations of certain functional sites of the ribosome. Dashed lines point to the invisible in the corresponding subunit’s projection proteins. The C-terminal tail of RP uS19 extending to the decoding site was not resolved on the 40S subunit model used here; its positioning is arbitrarily shown according to the data of the refs [20,21].

Here, we provide a review of recent reports on the functional consequences of replacements/deletions of particular RPs’ amino acid residues that are located at essential functional sites of the ribosome (Figure 1) and on currently accumulated data on the transport of RPs by extracellular vesicles. First, we discuss how specific amino acid residues of certain RPs contribute to the translation process, and on the other hand, we summarize data on intercellular transport of RPs by EVs and its biomedical significance. The summary does not pretend to be comprehensive, but it provides general ideas on current knowledge and points to the unresolved issues concerning this problem. Throughout the text, we use two nomenclatures for the designation of RPs. One of them (the newer one) is a universal nomenclature suggested in 2014 [48] and adopted among the scientists working in the fields of studying ribosome structure and translation process. Another (the older one) corresponds to the names of the RPs’ genes and still is used by researchers in other fields. For convenience, alternative names of RPs in all cases are given in parentheses.

## 2. Contribution of Specific RPs’ Amino Acid Residues to Translation and Its Regulation

### 2.1. Amino Acid Residues of RP uS19 Participating in Transmission of Conformational Changes via Intersubunit Bridges B1a and B1b/c

Intersubunit bridges B1a and B1b/c represent the connections between the small and the large ribosomal subunits within the whole ribosome that are universally conserved in all domains of life. In the eukaryotic 80S ribosome, B1a is formed by the small subunit RP uS19 (RP S15) and by the helix H39 of the large subunit 25S/28S rRNA, and B1b/c consists of the small subunit RP uS13 (RP S18) and of the large subunit RP uL5 (RP L11) [49,50,51]. RP uS19 has tight contacts with RP uS13 [15], which underlie close interrelations between alterations occurring with the components of each bridge. The bridges transmit conformation changes occurring at the small subunit during accommodation of the cognate aa-tRNA to the A site (mainly, rotation of the subunit head relative to its body) to the large subunit, affecting the arrangements of its functional centers [50]. These changes eventually alter positioning of the 3′-terminal part of the aa-tRNA in the large subunit so that it occurs in the PTC, where it can participate in the peptide bond formation (e.g., see [52]). In particular, rotation of the small subunit head results in the disruption of the B1a bridge [53,54,55].

A mutation study with yeasts [30] represents a fine investigation of the contribution of particular amino acid residues of RPs in translation in eukaryotes. Studying the effect of replacements of RP uS19 amino acid residues, which according to the structural data can interact with H38 or RP uL5, on translational properties of the ribosomes allowed the estimation of the residues significantly contributing to the rotational state of the ribosome. Translation properties of ribosomes with altered RP uS19 were examined in terms of their ability to form polysomes, to bind elongation factor eEF2 and to accommodate tRNA molecules at the A and the P sites. Changed ribosomal activity in these tests pointed to the implication of residues in the yeast RP uS19 positions 104–105 via their direct interaction with H38 in the bridge B1a and residues in the positions 112–114 indirectly via their interaction with RP uS13 that forms bridge B1b/c [30]. It should be noted here that some replacements in the RP uS19 amino acid residues potentially able to participate in the bridge B1b/c formation were lethal, which made investigation of ribosomes with such mutant protein impossible. Therefore, the question of what particular step of the small subunit assembly or translation was critically impaired in these cases remained unresolved.

### 2.2. Eukaryote/archaea-Specific Unstructured C-Terminal Tail of the RP uS19 (RPS15) at the Decoding Site

The occurrence of the C-terminal portion of the RP uS19 (RPS15) (amino acid residues in positions 130–145) in the decoding site of the mammalian 40S ribosomal subunit was initially detected in various site-directed cross-linking studies performed using the application of different mRNA analogues [6,8,9,11]. Later, the unstructured uS19 C-tail was visualized in the structures of model ribosomal complexes derived from high-resolution cryo-EM [20,21]. The C-tail of the eukaryotic RP uS19 has no homology in bacterial counterparts, and uS19 in the bacterial 30S subunit is located far from the decoding site, which made the above findings somewhat unexpected. The nature of the decoding process and the structure of the ribosomal decoding site were generally considered strongly conserved in all domains of life, which raised the special interest to the investigation of the functional role of the C-tail of RP uS19 in translation. 

**Figure 2:**
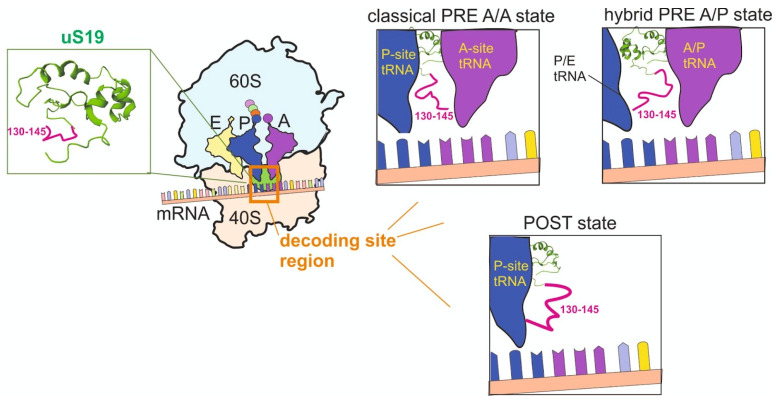
Functions of the unstructured C-terminal tail of RP uS19 shown in magenta on the structure of the protein in the human ribosome (PDB:4UG0) on the left. In the middle, a schematic picture showing the position of RP uS19 on the ribosome with mRNA and tRNA molecules at the A, P and E sites. On the right, simplified views representing the positioning of the uS19 tail relative to mRNA and tRNA molecules at the stage of codon recognition (classical PRE A/A state) after transpeptidation (hybrid A/P state) and after translocation (POST, posttranslocation state) according to [21].

The application of HEK293T cells producing mutant RP uS19 lacking the C-tail and analysis of the ribosomal material from these cells have revealed that the tail is not important for the assembly of the post-initiation 80S ribosome but is essential for the correct accommodation of incoming aminoacyl-tRNA (aa-tRNA) at the A site [26]. This step of the elongation cycle is an essential prerequisite enabling the participation of the cognate aa-tRNA in the peptide bond formation (see above). The molecular basis of interactions implicating different portions of the uS19 C-tail in the aa-tRNA accommodation became clear from a high-resolution cryo-EM study [21]. These interactions involve tRNA molecules at the A and P sites and the mRNA codon at the A site (Figure 2). The analysis of the structures of ribosomal complexes imitating consequent stages of the elongation cycle has shown that the positioning of the C-terminal uS19 tail dynamically changes during the cycle so that the contacts of certain amino acid residues become destroyed, while others, conversely, are set up [21]. Notably, the effect of replacements at certain positions of the C-terminal tail of RP uS19 on the decoding has also been observed in the fungus *Podospora anserina* [25], where they were reported to affect the accuracy of the stop codons’ recognition. However, in this case, molecular interactions mediating the effects of the replacements remained unknown. Interestingly, the effect of the interaction between the C-tail of RP uS19 and the mRNA codon at the decoding site on translation of mRNAs in living cells has also been demonstrated with the use of the PAR-CLIP approach [56].

It is worth noting here that the above fine investigations on the contribution of the C-tail of RP uS19 to the translation process have not yet provided a clearly formulated reply to the general question—which specific benefits have eukaryotes gained versus bacteria due to the emergence of the interactions of the uS19 tail at the decoding region? This question is interesting both as a fundamentally basic one and as an issue associated with chronic lymphocytic leukemia, which has been found to be associated with alterations in the C-tail of RP uS19 [57]. The relationship between the disease and mutations in the RP uS19 gene has been ascribed to the changes in cell life caused by altered function of their ribosomes [57].

### 2.3. Conserved Residues of RP uS3 (RPS3) at the Binding Site of the 3′-Terminal Portion of mRNA in the Ribosome Channel 

**Figure 3:**
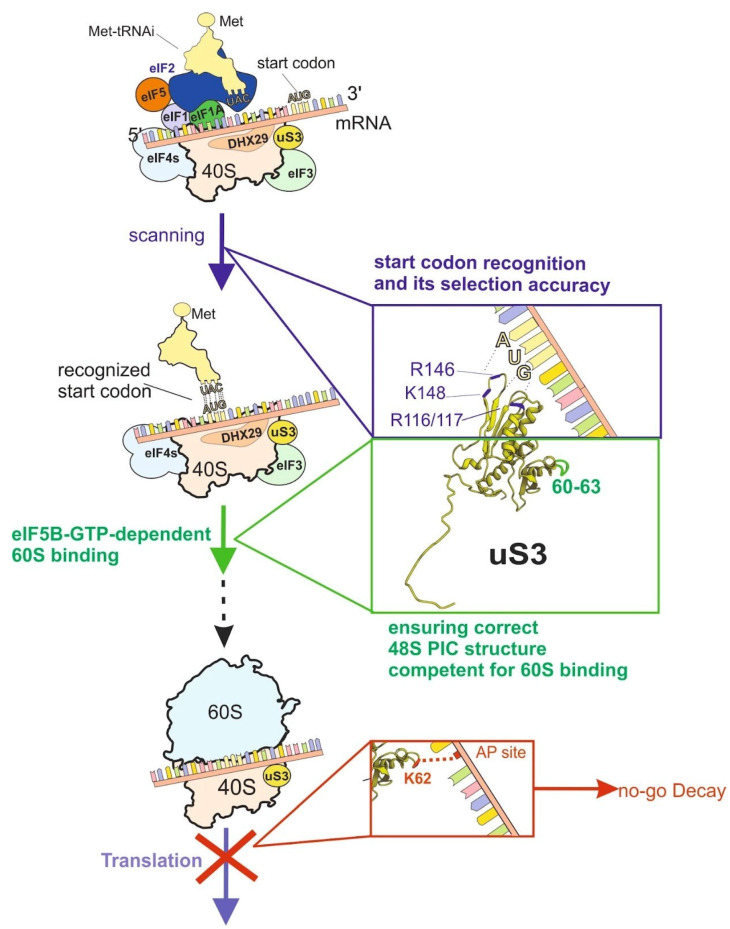
Functions of various RP uS3 amino acid residues. On the left, schematic representation of the 48S pre-initiation complex (PIC) before start codon recognition (upper image), after its recognition (image in the middle) and representation of the 80S ribosome assembled after completion of initiation (lower image). Translation on ribosomes is blocked when AP site in mRNA cross-links to the K62 residue of the RP uS3, which is a signal for disassembly of the ribosomal complex and degradation of the aberrant mRNA by the no-go-decay pathway. On the right, the structure of RP uS3 in the ribosome (PDB: 4UG0) with marked amino acid residues and comments on their functions.

The central region of the RP uS3 (RPS3) contains several conserved basic residues (R116, R117, R146, and K148 in the mammalian protein) that are believed to interact with the mRNA stretch between the entry site and the decoding area [28,58,59,60]. Residues R116/117 are located at the small subunit close to the mRNA entry pore, and mutation studies with yeasts have demonstrated that they are essential for stabilizing interactions between mRNA and the ribosome at the mRNA entry channel at the stages of the formation of pre-initiation and initiation complexes [58]. Disrupting the uS3-mRNA interactions in 40S subunits, where mutant uS3 has replacements for arginines at positions 116/117, leads to a decrease in the initiation efficiency and affects the discrimination of the correct start codon depending on its context [58]. These results have been generally supported in a very recent study on the effects of replacements for R116, R146 or K148 in the RP uS3 (RPS3) on translation in mammalian cells [28]. It has been found out that the roles of R146 and K148, by analogy with that of R116, relate to providing fidelity for the start codon selection. Intriguingly, these uS3 (RPS3) residues are crucial for the inhibition of cellular mRNA translation by the SARS-CoV-2 nonstructural protein NSP1 upon viral infection and their binding to the ribosome; furthermore, R116 is involved in mRNA degradation induced by the viral protein [28,61]. Taken together, it becomes clear that the translational roles of the basic RP uS3 (RPS3) residues contacting mRNA at the entry site and the neighboring part of the 40S subunit mRNA channel relate to the regulation of initiation, as could be expected from the structural data (Figure 3). At that, specific details of the implication of the uS3 (RPS3) residues have been revealed only by recent mutation studies. The discovery of the regulatory functions of these residues in the functioning of viral RNAs and in the fate of host mRNAs is a novel aspect of the involvement of the discussed uS3 residues in translation. 

### 2.4. Region around the K62 of the Mammalian RP uS3 (RPS3) That Cross-Links to Single-Stranded DNAs and RNAs Bearing Aldehyde Groups

Region 55–64 of the mammalian RP uS3 (RPS3) is exposed at the solvent side of the 40S subunit near the mRNA entry pore. It possesses an ability, unique among RPs, to selectively cross-link to single-stranded model RNAs and DNAs containing aldehyde groups, such as those with oxidized 3′-terminal ribose to dialdehyde [13,62] or an abasic site [63,64,65]). It is a well-known fact that aldehydes react with aliphatic amines (in proteins with amine groups of lysines and arginines) to form Schiff bases, and the ability of the RP uS3 55–64 to cross-link to aldehyde-containing nucleic acids is likely provided by the K62. This feature is apparently implicated in ribosome-based mRNA quality control, in the course of which damaged mRNA cross-links to RP uS3 (RPS3) via the abasic site, leading the translation to stall and making the resulting ribosomal complex a target for degradation via the No-go decay pathway [64,65] (Figure 3). Similarly to the previously discussed C-tail of RP uS19 (RPS15), the role of this RP uS3 (RPS3) region in translation has been explored using cells that produce FLAG-tag-labeled uS3 with alanine substitutions at positions 60–63 [23]. The mutant protein was observed exclusively in 40S subunit fractions but not in 80S ribosomes and polysomes derived from the above cells, which was the basis for the conclusion that RP uS3 residues 60–63 are critically important for maintaining the correct structure of the 48S preinitiation complex (PIC) competent for the eIF5A-dependent binding of the 60S subunit and starting the first elongation cycle (Figure 3). The implication of the RP uS3 tetrapeptide 60-GEKG-63 in the organization of the PIC has been ascribed to its interaction with initiation factor eIF3 subunit eIF3j [23]. This subunit occupies the mRNA channel before start codon recognition [66] to prevent premature loading of the coding sequence. Alternatively, the RP uS3 tetrapeptide might interact with helicase DHX29 (which assists translation of mRNAs with stable secondary structures in the 5′-untranslated region), whose binding site on the 40S subunit overlaps with that of eIF3 [67,68]. Thus, in the case of the tetrapeptide in positions 60–63 of the mammalian RP uS3, investigation of the role of particular amino acid residues of an RP has revealed completely novel knowledge concerning its contribution to the translation process that could hardly have been predicted from the structural data.

### 2.5. Conserved Motif YxxPKxYxK of the eS26 (RPS26) at the mRNA Exit Site

The close proximity of the RP eS26 (RPS26) to the mRNA stretch upstream of the E site codon on the mammalian ribosome has been observed in site-directed cross-linking studies carried out in two groups with different types of mRNA analogues [6,9]. The cross-linking site of mRNA analogues has been mapped to the region comprising amino acid residues in positions 60–71 of the human RP eS26, which remains conserved in the eukaryotic eS26 (RPS26) family motif YxxPKxYxK [12]. Thus, it was expected that the role of the mRNA-eS26 contact primarily concerns the maintenance of the path of mRNA from the decoding area to the exit site [12]. In addition, it has been assumed that the above eS26 motif takes part in the interaction of the 40S subunit with the eukaryote-specific initiation factor eIF3, whose binding area on the subunit overlaps with the above eS26 region [22]. 

**Figure 4:**
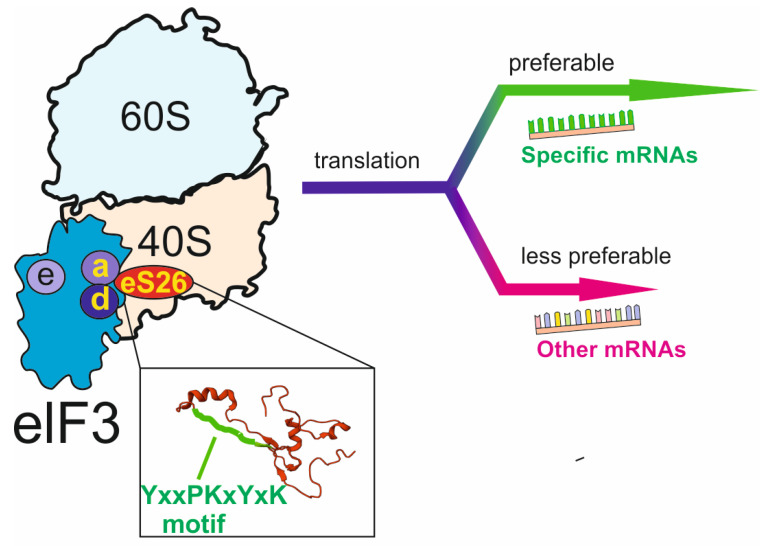
Functions of the YxxPKxYxK motif of RP eS26 in translation. A schematic representation of the ribosome containing bound eIF3 at the elongation stage of protein synthesis (mRNA and tRNAs are not shown) and a zoomed structure of RP eS26 in the ribosome (PDB: 4UG0). Subunits eIF3a and/or eIF3d interact with the YxxPKxYxK motif, which contributes to the binding of eIF3 to the ribosome and thereby promotes the eIF3e-dependent selective translation of specific mRNAs.

The examination of the role of the YxxPKxYxK motif in translation has shown that even simultaneous replacement of all conserved amino acid residues with alanines does not critically impair translation in yeast [69] and human [27] cells. These somewhat unexpected results implied that the above motif does not contribute significantly to the bulk translation, and interactions of the mRNA RP eS26stretch upstream from the E site codon with either are not principally important for translation process as a whole or do not exist at all. More detailed analysis performed with human cells has revealed that the simultaneous replacement of all five conserved residues of the YxxPKxYxK motif in the RP eS26 leads to the accumulation of light polysomes versus heavy ones [27]. Moreover, these light polysomes are enriched with eIF3 that remains associated with ribosomes after completing initiation of translation [27]. It is known that the initiation factor eIF3 subunit eIF3e remaining bound at the elongating ribosomes promotes a selective translation of certain mRNAs [70]. Indeed, translation of these specific mRNAs was altered in ribosomes containing the mutant RP eS26, and it has been concluded that the YxxPKxYxK motif is implicated in the fine regulation of translation of selected mRNAs to maintain the balance of proteins currently required for cell life [27]. Analysis of available PIC structures in the above study allowed the authors to suggest that the RP eS26 motif interacts with the factor subunits eIF3a and/or eIF3d and thereby facilitates the recruitment of the whole eIF3 containing eIF3e (Figure 4). Thus, in the case of the RP eS26 YxxPKxYxK motif, only one (and the less expected) of two potential roles in translation deduced from the structural and biochemical data has been confirmed and substantiated. The above mutation studies with RP eS26 also provided an explanation as to why the YxxPKxYxK motif is conserved in the eukaryotic RP eS26 family but not in the archaeal one. The role of the motif is associated with its interactions with eIF3, and this initiation factor exists and operates only in eukaryotes but not in archaea.

### 2.6. “GGQ Minidomain” of eL42 (RPL36a) at the Ribosomal E Site

According to a site-directed cross-linking study [14] and information from structures of mammalian 80S ribosomal complexes derived via high resolution cryo-EM (e.g., see [71,72,73], the 3′-terminus of deacylated tRNA bound at the P site in the hybrid P/E state (the state in which the anticodon stem-loop remains bound at the P site while the acceptor stem arrives at the E site) contacts K53 of RP eL42 (RPL36a). Near this residue, in positions 49–51, there is a tripeptide GGQ conserved in the eukaryotic RP eL42 family [14]. It is rare for RPs, but in the polypeptide chain of the release factor eRF1, this universally conserved motif plays a critically important role, being responsible for inducing the hydrolysis of the ester bond between peptidyl residue and the P site tRNA in the PTC at translation termination [74,75,76,77,78]. All this was the basis for a hypothesis on the implication of the eL42 mini-region comprising the GGQ tripeptide in hydrolysis of the peptidyl-tRNA at the P site during termination of translation and in promoting transfer of the peptidyl moiety to the aminoacyl-tRNA at the A site during elongation [22] (note that eL42 was there referred as eL44).

**Figure 5:**
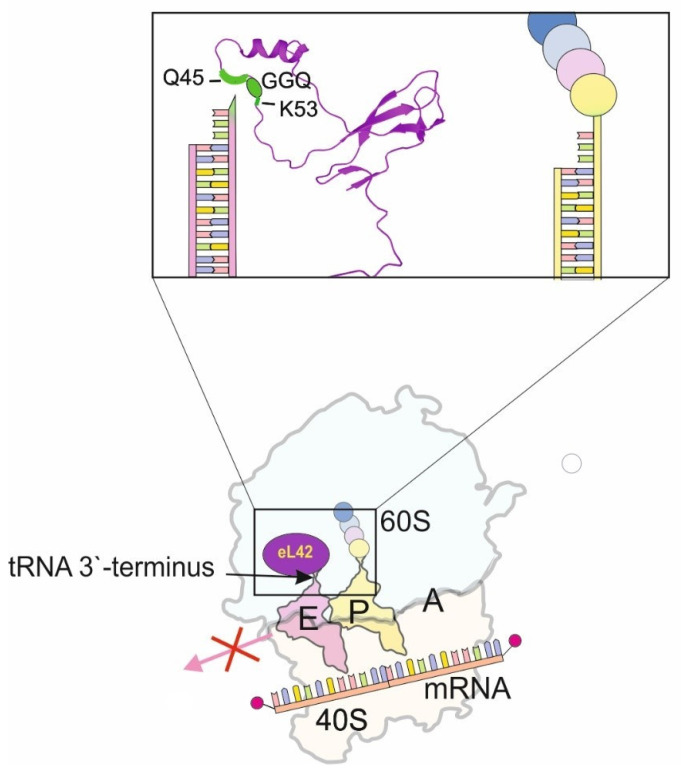
Function of the “GGQ” minidomain of RP eL42. Schematic representation of the 80S ribosomal complex containing mRNA, peptidyl-tRNA at the P site and deacylated tRNA at the E site. In the rectangle, there is the structure of RP eL42 in the ribosome (PDB: 4UG0) with marked the GGQ motif, amino acid residues in the positions 45–53 (human numbering) around it and a schematic representation of the acceptor stems of the E site and P site tRNA molecules relative to the protein. Interaction of the “GGQ” minidomain of RP eL42 with the 3′-terminus of the E site tRNA ensures its release at the respective step of the elongation cycle.

Experimental data on the functional role of the GGQ region of the RP eL42 in translation have been obtained in mutation studies with yeast [24]. Replacements of certain amino acid residues in the above region, primarily of the K55 corresponding to K53 in human eL42 (which contacts the 3′-OH terminus of the P/E site-bound deacylated tRNA, see above, and of the Q residue in the GGQ motif) considerably affected cell life. They were either lethal to yeasts or resulted in a significant decrease in the translational activity of the ribosomes containing the mutant eL42 examined in vitro by their ability to perform the poly(U)-directed synthesis of poly(Phe) and transfer of a labeled acetylated amino acid residue from a peptidyl-tRNA analogue to puromycin [24]. All this indicated the importance of the GGQ region of eL42 for elongation of peptide chain, and its role has been attributed to the direct involvement in the peptidyl transferase reaction. However, several unresolved issues regarding the implication of the eL42 GGQ region in translation still remain unclear. The effect of the amino acid replacements on the translation of cellular mRNAs in vivo has not yet been examined. Moreover, it is difficult to understand how the GGQ region of RP eL42 could be implicated in peptide bond formation. The question arises from the careful analysis of the atomic models of human ribosomal complexes representing classical pre-translocation, hybrid pre-translocation and post-translocation states (PDB accession numbers 6Y0G, 6Y57 and 6Y2L, respectively) [21]. The analysis shows that the GGQ region is located far (>45 Å) from all 28S rRNA nucleotides that are directly involved in PTC formation in all examined ribosome states. Thus, there is room for alternative explanations for the ways by which the GGQ region of eL42 is implicated in the elongation of peptide chains. Considering that this region is located in close proximity to the 3′-end of deacylated tRNA at the P/E state (see above), one can assume that alterations in or around the GGQ tripeptide of RP eL42 impedes dissociation of tRNA from the E site and thereby impairs elongation (Figure 5).

### 2.7. Hydroxylated H39 of the RP uL15 (RPL27a) at the Region Close to PTC

**Figure 6:**
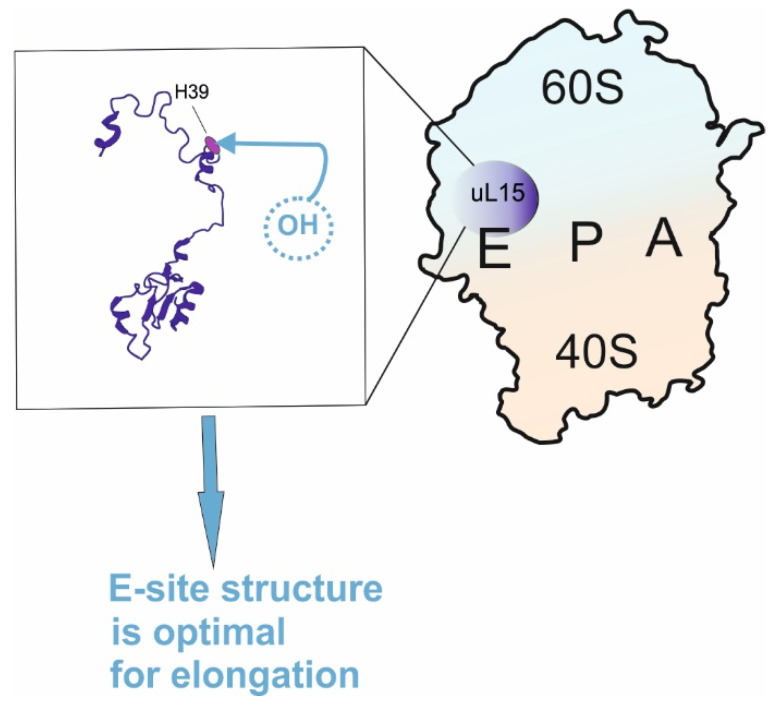
Function of the hydroxylated H39 in RP uL15 (the protein structure shown in the left with marked H39 was extracted from the 60S subunit structure, PDB: 4UG0).

Interest in the examination of the contribution to translation of the specific amino acid residues of RPs concerns not only those contacting ribosome ligands according to structural data, but also specific post-translational modifications of RPs. In this vein, it is known that several RPs (namely, uL2 (RPL8), uL15 (RPL27a), and uS12 (RPS23)) within the ribosome are selectively hydroxylated at the specific amino acid residues by so-called ribosomal oxygenases [79,80]. These residues are located near the functional sites of the ribosome (uL2 (RPL8) close to the PTC, uL15 (RPL27a) near the CCA-terminus of the E site-bound tRNA and uS12 (RPS23) near the decoding site), which raises the question of the functional significance of the above post-translational modifications for translation. The relevant study was performed with human RP uL15, in which mutations were introduced at position 39, corresponding to the site of hydroxylation of the H39, or at the neighboring position 40 [29]. In the cells producing the mutant protein derived from the ability of the specific hydroxylation, its content in polysomes was less than that of the wild type RP uL15. On the other hand, the mutation did not affect incorporation of the RP uL15 into the 60S subunits or into the 80S ribosomes; however, it caused specific changes to the repertoire of translated mRNAs [81]. These findings indicate the significance of the hydroxylation of RP uL15 at H39 for translation elongation in living human cells. The contribution of H39 and H40 to the ribosomal translational activity was attributed to maintaining its structure near the E site optimal for the functioning [29] (Figure 6). The implication of these residues in ribosome structure stabilization was likely mediated by a hydrogen bond between the N atom of the imidazole ring of H40 and the hydroxyl group of H39. For all that, details of the implication of hydroxylation of H39 in the elongation process remain unclear. Further investigations are required in order to find out which particular step(s) of the elongation protein synthesis depend(s) on the hydroxylated H39 and which molecular interactions in the ribosomal functional sites provide the contribution of the modification to the elongation process.

## 3. Transport of RPs by Extracellular Vesicles and Its Functional Consequences

RPs are essentially involved in cell life both via their functioning as constituents of the ribosome and via their extra-ribosomal functions. Therefore, one could expect that if ribosomal proteins can be sorted to an extracellular vesicle (EV) as passengers, transfer of some RP from one cell to another can cause significant alterations in the receptor cell. Indeed, more than a third of 80 ribosomal proteins have been found together with other RNA-binding proteins within EVs of various origin, including exosomes (e.g., see [37]), and this number is constantly increasing. RPs have been found in EVs produced by various normal cells [38,39] and, even more often, by malignant ones [36,43,44,46]. It should be taken into account that available data on RPs as passengers of exosomes are somewhat contradictory, in particular regarding exosomes from human colon adenocarcinoma and glioblastoma cancer cell lines. Notably, certain RPs, namely, uS3 (RPS3) and eS8 (RPS8) together with several other RNA binding proteins, which have been considered typical components of exosomes, were not observed any more following purification of exosomes by high-resolution iodixanol gradients [42]. Nevertheless, the purification approach suggested in the above study did not become conventional and was not commonly applied in the works from other groups published afterwards. One also cannot exclude the fact that the described purification procedure is somewhat excessive and potentially leads to the loss of some passenger proteins. 

Numerous data suggest that specific RPs are selectively packed (sorted) into certain EVs; in particular, RPs often are considerably enriched in EVs compared to the respective cell lysates. Examples of such enrichment have been observed in (i) eS24 (RPS24) and eL34 (RPL34) in EVs from nonmineralizing and mineralizing human osteoblast cells [36], (ii) in uS3 (RPS3) in exosomes from mouse embryonic fibroblasts [41], (iii) in eL13 (RPL13) and eL14 (RPL14) (together with other RPs) in exosomes from pooled plasma samples of imatinib-resistant chronic myeloid leukemia patients [44] and (iv) in uL1 (RPL10A), uS13 (RPS18), eS30 (RPS30), eL14 (RPL14) and uS2 (RPSA) in exosomes from ischemia-challenged epicardial adipose tissue-derived stem cells [40]. Various populations of EVs from the same cells can be differently enriched in particular RPs, which was detected in small and large EVs from various breast cancer cell lines [46]. Moreover, sometimes RPs were detected only in EVs and not in fluids from which the EVs had been isolated, as was reported with eS6 (RPS6) and eS17 (RPS17) in EVs from bovine ovary follicular fluid [39]. All this implies that specific RPs are selectively sorted in certain EVs. 

It has long been known that the level of many RPs significantly increases in malignant cells compared to non-malignant ones (e.g., for review see [82,83]), and therefore, it is obvious to suggest that the increased content of RPs in EVs is associated with cancer progression and/or metastasis. Interestingly, the relationships between RPs and cancer with different RPs in some cases are opposite. For example, with glioblastoma, eS6 (RPS6), eS27 (RPS27), uS8 (RPS15A) and eL34 (RPL34) are known as oncogenic [84,85,86,87,88,89], whereas uL18 (RPL5), uS17 (RPS11), uS9 (RPS16) and uS13 (RPS18) are found to have a tumor-suppressive function [90,91]. Also well-known is the involvement of RP uS3 (RPS3) [92,93,94] and uS2 (RPSA) [95,96,97] in the invasion and metastasis of tumor cells (note that uS2 (RPSA) may work outside the ribosome as a laminin receptor). The oncogenic properties of certain RPs and their enrichment in EVs from specific cancer cells make them potential markers of tumors and of the prognosis of their progression and metastasis.

Little is known about the functional assignment of the RPs as a cargo of EVs, including exosomes. The main idea of the role of exosomes (and possibly, other EVs) in an organism’s life is that they transfer biomolecules from one cell to another in order to change the phenotype of the receptor cell in a programmed way [98,99,100]. Such a role of RPs, together with that of other RNA-binding proteins, might be associated with intercellular transport of RNAs by EVs. Indeed, 20 RNA-binding proteins capable of forming complexes with exosomal RNAs in exosomes from human epithelial lung cells have been identified, and among them were three RPs, uL11 (RPL12), uS3 (RPS3) and uS13 (RPS18) [38]. But the most intriguing question related to the idea on the role of RPs as passengers of EVs affecting the phenotype of the receptor cell concerns the ways by which these proteins are often associated with carcinogenesis. Communication between cells in the organism via exosomes and other EVs possibly contributes to the dissemination of malignant cells in the course of metastasis and/or to malignant transformation of the cell. Remarkably, several examples of evidence supporting this assumption have been recently obtained. 

Cis-diamminedichloroplatinum(II) (cisplatin) is a well-known anti-tumor agent, and resistance of some cancer cell lines to this drug is associated with RP uS3 (RPS3), which activates the NF-κB pathway due to an extra-ribosomal function of the protein as a subunit of the NF-κB complex [92]. Recently, it has been shown that delivery of RP uS3 from a cisplatin-resistant gastric cancer cell line to the sensitive one by exosomes results in the change of the receptor cells phenotype to the resistant one mediated by the PI3K-Akt-cofilin-1 signaling pathway, which is involved in the mitochondrial apoptosis of cisplatin-resistant gastric cancer cells [47]. Exosomes carrying RP uS3 from the resistant cells decreased translocation of cofilin-1 to mitochondria and thereby decreased apoptosis in the cisplatin-sensitive cells. The effect was also caused by the elevation of the RP uS3 level in the cell without the action of exosomes, and its excessive production directly in the cisplatin-sensitive cells led to the same phenotypic alterations as those caused by the exosome-transported protein [47].

Other examples of malignant reprogramming of cells induced by RPs as exosomal cargo mainly concern data obtained from glioblastoma (GBM) tumor cells (summarized in [45]). Briefly, exosomes secreted by GBM cells were found to change the phenotype of neural stem cells to a tumor type [101]. Such EVs produced by glioblastoma stem cells (GSC) are able to reprogram non-GSC stem cells into malignant ones [102]. GBM EVs can also transform the phenotype of normal astrocytes into tumor-supporting one [103,104]. Notably, there is some indication that in certain cases, whole extrinsic ribosomes can reprogram receptor cells. These ribosomes, when derived from GBM cells, induce the formation of so-called ribosome-induced cancer cell spheroids and stem-cell-like phenotypes; the latter is associated with the expression of RP eS6 (RPS6) and its phosphorylation [45]. The ability of RP eS6 to promote induction of stem-cell-like phenotypes is regarded as a new extra-ribosomal function of this RP [45]. All this demonstrates that exosomes from GBM cells have the ability to reprogram receptor cells. On the other hand, such properties could be peculiar to extrinsic ribosomes and RP eS6. Thus, one can assume that the ability of extrinsic ribosomes and RP eS6 to change the phenotype of the receptor cell might be mediated by exosomes or other EVs carrying ribosomes and RP eS6 as cargo.

Finally, the most recent study shows that ischemia promotes a significant increase in the level of secretion of certain RPs (primarily, of uL1 (RPL10A), uS13 (RPS18), eS30 (RPS30), eL14 (RPL14) and uS2 (RPSA)), and they become considerably enriched in the exosomes of ischemia-challenged epicardial adipose tissue-derived stem cells [40]. Moreover, detailed proteomic analysis of the exosomal RPs from these cells has led to the conclusion that these proteins drive unique phenotypes of the above cells populations [40], which can be regarded as an indication for reprogramming abilities of exosomes carrying the respective protein passengers.

## 4. Conclusions

The recent achievements concerning the first and the best studied extreme of the knowledge on RPs’ functions in translation are impressive. Evidently, biochemical, structural and mutation studies together have provided to date clearly supported ideas on the participation of certain amino acid residues of RPs in particular steps of translation in eukaryotes. This knowledge has a fundamental significance since it concerns understanding driving forces of translation, one of the essential processes of cell life. As well, it is important to biomedicine in areas related to ribosomopathies, pathologies caused by mutations in genes coding for RPs and the considerably increasing risk of cancer. A number of such congenital diseases originating mainly from alterations in RPs leading to defects of the ribosome assembly are well-known (Diamond–Blackfan anemia, Schwachman–Diamond syndrome, dyskeratosis congenita, cartilage hair hypoplasia, Treacher Collins syndrome, etc. [105,106,107]). Additionally, impairment of the ribosome assembly caused by mutations in ribosomal protein genes can be associated with carcinogenesis [108]. Less studied are disorders related to RP mutations that do not hamper ribosome assembly but affect translation by the ribosome containing the altered RP. Examples of such disorders concern mutations in the C-terminal tail of RP uS19 (RPS15) [57] and in RP uL16 (RPL10) (reviewed in [109]). One cannot exclude the possibility that new ribosomopathies caused by such mutations will be further discovered. Despite remarkable progress in understanding the roles of certain amino acid residues of RPs in translation, not all points are fully settled, and several questions remain to be further clarified. It remains largely unknown which particular interactions mediate (i) participation of the tetrapeptide 60-GEKG-63 of RP uS3 (RPS3) in maintaining the correct structure of the 48S preinitiation complex capable of the 60S subunit binding, (ii) implication of the motif YxxPKxYxK of RP eS26 (RPS26) in eIF3e-dependent regulation of specific mRNAs translation, (iii) involvement of the GGQ mini-region of RP eL42 (RPL36a) and hydroxylated H39 of RP uL15 (RPL27a) in the elongation of peptide chain. Notably, the translational role of selective hydroxylation of specific amino acid residues in RPs uL2 (RPL8) and uS12 (RPS23) to our knowledge has not been yet studied. All these issues remain to be clarified. Finally, the currently accumulated information on amino acid residues of RPs at the keystone functional sites of the ribosome that considerably contributes to the translation process is yet not comprehensive, and a number of potentially important ones have not yet been examined. 

The opposite “edge” of the knowledge on the RPs function concerning their extracellular transport by EVs rapidly grows. The very recent data obtained from different kinds of EVs secreted by various types of cells and carrying numerous RPs demonstrate that these proteins are rather common and important representatives of the EVs’ cargo. Moreover, first lines of experimental evidence have been obtained indicating the ability of exosomes carrying particular RPs to reprogram receptor cells, causing alteration of their phenotype. Importantly, such alterations can make them malignant or increase already-existent malignant properties (the ability for proliferation, insensitivity to anti-tumor drugs, etc.). These data have both fundamental and biomedical significance; the latter might be also associated with well-known oncogenic features of a number of RPs whose level often is dramatically increased in tumor cells compared to normal ones. For all that, in the field of extracellular transport of RPs by EVs to date, there are more questions than answers. Namely, it is unclear to what extent reprogramming features of EVs carrying RPs as a cargo are common (considering the nature of secreting and receptor cells, type of EVs and each RP). It remains unknown how RPs are specifically sorted into EVs and which molecular partners mediate their packing. Additionally, ways by which RPs transported to a receptor cell change its phenotype are obscure, and it is unclear how the changes contribute to malignant transformation of the cell or increase malignant properties of tumor cells. All this forms the next frontiers for further investigations.

## Data Availability

No new data were created or analyzed in this study. Data sharing is not applicable to this article.
